# HMGN2 and Histone H1.2: potential targets of a novel probiotic mixture for seasonal allergic rhinitis

**DOI:** 10.3389/fmicb.2023.1202858

**Published:** 2023-10-06

**Authors:** Lisha Li, Xueyi Wen, Yiyi Gong, Yuling Chen, Jiatong Xu, Jinlyu Sun, Haiteng Deng, Kai Guan

**Affiliations:** ^1^Department of Allergy, State Key Laboratory of Complex Severe and Rare Diseases, Peking Union Medical College Hospital, Chinese Academy of Medical Science and Peking Union Medical College, Beijing, China; ^2^Medical Research Center, Peking Union Medical College Hospital, Chinese Academy of Medical Science and Peking Union Medical College, Beijing, China; ^3^Ministry of Education (MOE) Key Laboratory of Bioinformatics, Center for Synthetic and Systematic Biology, School of Life Sciences, Tsinghua University, Beijing, China; ^4^Tsinghua University-Peking University Joint Center for Life Sciences, Beijing, China

**Keywords:** allergic rhinitis, probiotics, randomized placebo-controlled trial, proteomics, high-mobility group nucleosome-binding domain-containing protein 2 (HMGN2), Histone H1.2

## Abstract

**Background:**

Allergic rhinitis (AR) is a common nasal inflammatory disorder that severely affects an individual's quality of life (QoL) and poses a heavy financial burden. In addition to routine treatments, probiotic intervention has emerged as a promising strategy for preventing and alleviating allergic diseases. The main objective of this study was to determine the effect of a novel multi-strain probiotic mixture on AR symptoms and investigate potential targets underlying the probiotic intervention.

**Methods:**

A randomized, double-blind, placebo-controlled clinical study was conducted on AR patients who were allergic to autumnal pollens (*n* = 31). Placebo or a novel probiotic mixture, composed of *Lactobacillus rhamnosus (L. rhamnosus)* HN001, *L. acidophilus* NCFM, *Bifidobacterium lactis (B. lactis)* Bi-07, *L. paracasei* LPC-37, and *L. reuteri* LE16, was administered after 2 months. The therapeutic efficacy was evaluated by a symptom assessment scale. Before and during the pollen season, blood samples were collected, and peripheral blood mononuclear cells (PBMCs) were isolated for further tandem mass tags (TMTs)-based quantitative proteomic analyses. Potential targets and underlying pathological pathways were explored using bioinformatics methods.

**Results:**

During the pollen season, the rhinoconjunctivitis symptom score of participants who were administered probiotics (probiotic group, *n* = 15) was significantly lower than those administered placebo (placebo group, *n* = 15) (*P* = 0.037). The proteomic analyses identified 60 differentially expressed proteins (DEPs) in the placebo group, and subsequent enrichment analyses enriched a series of pathways and biological processes, including signaling pathways of inflammation, coagulation cascade, lipid, carbohydrate and amino acid metabolic pathways, and transcription and translation processes. Least Absolute Shrinkage and Selection Operator (LASSO) regression extracted five main elements, namely, GSTO1, ATP2A2, MCM7, PROS1, and TRIM58, as signature proteins. A total of 17 DEPs were identified in the probiotic group, and there was no pathway enriched. Comparison of DEPs in the two groups revealed that the expression levels of the high-mobility group nucleosome-binding domain-containing protein 2 (HMGN2) and Histone H1.2 presented an opposite trend with different interventions.

**Conclusion:**

Our data showed that AR symptoms alleviated after treatment with the novel multi-strain probiotic mixture, and the proteomic analyses suggested that HMGN2 and Histone H1.2 might be targets of probiotic intervention for seasonal AR.

## Introduction

Allergic rhinitis (AR) is a common nasal inflammatory disorder, and it is characterized by pruritus, sneezing, rhinorrhea, and nasal obstruction (Bousquet et al., 2008). Although AR is not a fatal illness, it severely affects an individual's quality of life (QoL) and poses a heavy financial burden (Dierick et al., 2020). Furthermore, AR underlies multiple complications such as asthma and atopic dermatitis (Togias, 2003; Gabryszewski and Hill, 2021). In China, it is estimated that 17.6% of the population is affected by AR, and the prevalence has been progressively increasing (Wang et al., 2016; Tong et al., 2020). China's nationwide map of AR shows that grass and tree pollen are one of the most common types of aeroallergens, and related cases are concentrated in the northern area (Wang et al., 2018).

The classical allergic reaction of AR is the predominance of immunoglobin E(IgE)-mediated type 2 immune response (Zhang et al., 2021). The new antigen is captured by antigen-presenting cells (APCs), generally dendritic cells (DCs) and macrophages. APCs process antigen to allergenic peptides, which are presented to cognate naïve T cells under the presence of interleukin-4 (IL-4), IL-13, and other cytokines, provided by mast cells, basophils, and other cell types in earlier processes. Cognate naive T cells are then activated and differentiated into T helper type 2 (T_H_ 2) cells, engaging with cognate B cells and inducing B cells to undergo an IgE class switch (Galli and Tsai, 2012). The released IgE molecules subsequently bind to high-affinity receptors (FcεRI) expressed on the surface of mast cells and basophils located in the nasal mucosa. In addition to interplaying with B cells, T_H_ 2 cells release a wide spectrum of cytokines, such as IL-4, 5, 10, and 13, exacerbating T_H_2-dominated inflammation reactions. When the sensitized individual re-encounter the allergen, it binds to allergen-specific IgE on mast cells and basophils and leads to the cross-linking of IgE and FcεRI. Once many dimeric or higher-order oligomeric receptor molecules are cross-linked, mast cells are activated and release preformed mediators, leading to canonical symptoms of AR (Knol, 2006; Palmer et al., 2017).

Probiotics are defined as live microorganisms that confer the desirable effects to the host when provided with an adequate amount (F. Who, 2011). Recently, supplementation with probiotics emerges as a novel intervention in the prevention of allergic diseases (Plaza-Diaz et al., 2019; Jamalkandi et al., 2021). A body of clinical trials has been carried out to explore the role of this non-pharmaceutical agent in the treatment of AR (Ishida et al., 2005; Xiao et al., 2006a,b; Tamura et al., 2007; Ivory et al., 2008; Gotoh et al., 2009; Kawase et al., 2009; Nishimura et al., 2009; Koyama et al., 2010; Nagata et al., 2010; Wassenberg et al., 2011; Singh et al., 2013; Costa et al., 2014; Dölle et al., 2014; Dennis-Wall et al., 2017; Jalali et al., 2019; Kang et al., 2020). However, highly heterogeneous results were obtained, which may be caused by different strains of probiotics, dose, and duration. The potential mechanism underlying probiotic treatment may attribute to its role in the correction of T_H_1/T_H_2 imbalance by targeting cytokines or immune cells (Luo et al., 2022). For example, *Lactobacillus rhamnosus (L. rhamnosus)* HN001 is a well-characterized probiotic strain often used in probiotic products. In addition to surviving in adverse gastrointestinal conditions and adhering to the intestinal mucosa, this probiotic strain possesses strong immunomodulatory activities. For example, the bacterial strain can invoke IFN-γ, IL-2, and IL-10 secretion during the ongoing Th2-type immune response (Cross et al., 2002). *L. acidophilus* NCFM is another common strain in the probiotic industry. The ability to modulate immune responses and improve immune functions is demonstrated in the previous study. For example, it can affect the function of immune cells and the production of cytokines such as IL-12 and tumor necrosis factor (TNF)-α (Mathiesen et al., 2019; Wang et al., 2019). Moreover, *L. acidophilus* NCFM can promote IL-4 and IL-10 production by affecting the activity of T cells and DCs (Konstantinov et al., 2008). There are some types of probiotics, such as *Bifidobacterium lactis* (*B. lactis*) Bi-07 (Childs et al., 2014), *L. paracasei* LPC-37, and *L. reuteri* LE16, that show great potential in clinical treatment through their immunomodulatory effects but the underlying mechanism remains a mystery. The former clinical observations indicate that the effect of probiotics on AR is not clear and inconsistent; significant challenges still exist in designing efficient probiotic therapies. In addition, detailed mechanisms of the probiotic action have not been completely elucidated. In this study, we monitored the effect of a novel multi-strain probiotic mixture on relieving symptoms in subjects allergic to autumnal pollens. The selected five strains include *L. rhamnosus* HN001, *L. acidophilus* NCFM, *B. lactis* Bi-07, *L. paracasei* LPC-37, and *L. reuteri* LE16, with the function of immune modulation. Furthermore, to explore underlying immunological mechanisms of the seasonal AR and potential targets of the novel probiotic mixture, tandem mass tags (TMTs)-based quantitative proteomics was conducted on peripheral blood mononuclear cells (PBMCs), and subsequent bioinformatics analyses including enrichment analyses, protein–protein interaction (PPI), and least absolute shrinkage and selection operator (LASSO) regression were applied.

## Materials and methods

### Subjects

A total of 31 AR patients who are allergic to autumnal pollen allergens were recruited from the outpatient allergy clinic of Peking Union Medical College Hospital. AR was diagnosed according to the diagnostic criteria proposed by the World Health Organization (WHO) workshop (Bousquet et al., 2008) and Chinese experts from Allergic Rhinitis Committee (Zhang, 2015). Inclusion criteria were as follows: (i) patients diagnosed with AR, (ii) adult men and women (≥ 18 years old), (iii) patients with a history of classic rhinoconjunctivitis symptoms during the autumn pollen season for at least 2 years and without symptoms in other seasons, (iv) positive intradermal skin test (wheal ≥ 10 mm) to grass and tree pollens spread in autumn (*Mugwork, Japanese Hop, Firebush, Cocklebur, Daisy, Dandelion, Plantain, Goosefoot*, and *Common Goldenrop*) (Allergen Manufacturing Laboratory of Peking Union Medical College Hospital, Beijing, China), and (v) corresponding serum allergen-specific IgE (sIgE) of ≥ 0.7 KUA/L (ImmunoCAP, ThermoFisher Scientific Inc. Waltham, MA, USA). Exclusion criteria were as follows: (i) patients with a history of asthma, immunodeficiency, chronic infection, or tumor; (ii) pregnancy or lactation, (iii) patients with a history of allergen immunotherapy, (iv) regular consumption of probiotic-supplemented foods, and (v) administration of antibiotics, immunosuppressive medications, monoclonal antibodies, or a large dose of corticosteroids in a month preceding this study. This study was approved by the Medical Ethics Committee of the Peking Union Medical College Hospital (JS-1649). Before enrollment, all participants were provided with written informed consent.

### Probiotics and placebo

The probiotics used in this study were a multi-strain mixture that composes of 3 × 10^9^ colony-forming units (CFU) *L. rhamnosus* HN001, 2 × 10^9^ CFU *L. acidophilus* NCFM, 3 × 10^9^ CFU *B. lactis* Bi-07, 1 × 10^9^ CFU *L. paracasei* LPC-37, and 1 × 10^9^ CFU *L. reuteri* LE16. Moreover, the products are synthesized by a pharmaceutical company that is specialized in probiotic-fortified food products. Participants were given either the probiotic powder or the placebo. The active sample consisted of the probiotic mixture (10^10^ CFU/g) and galactooligosaccharides, while the placebo contained only galactooligosaccharides. Products were provided in 2 g small packs and were identical in flavor and appearance.

### Study design

This was a randomized, placebo-controlled, double-blind study conducted from May 1, 2017 to October 15, 2017. In May and June, AR patients meeting the inclusion criteria were enrolled in this study. The sample size was chosen according to the criteria of phase 1 clinical trials. Participants were allocated into two groups (namely, the probiotic group and the placebo group) using block randomization. Participants were administered probiotics or a placebo of 2 g pack each day starting from July 1, 2017.

**Figure 1:**
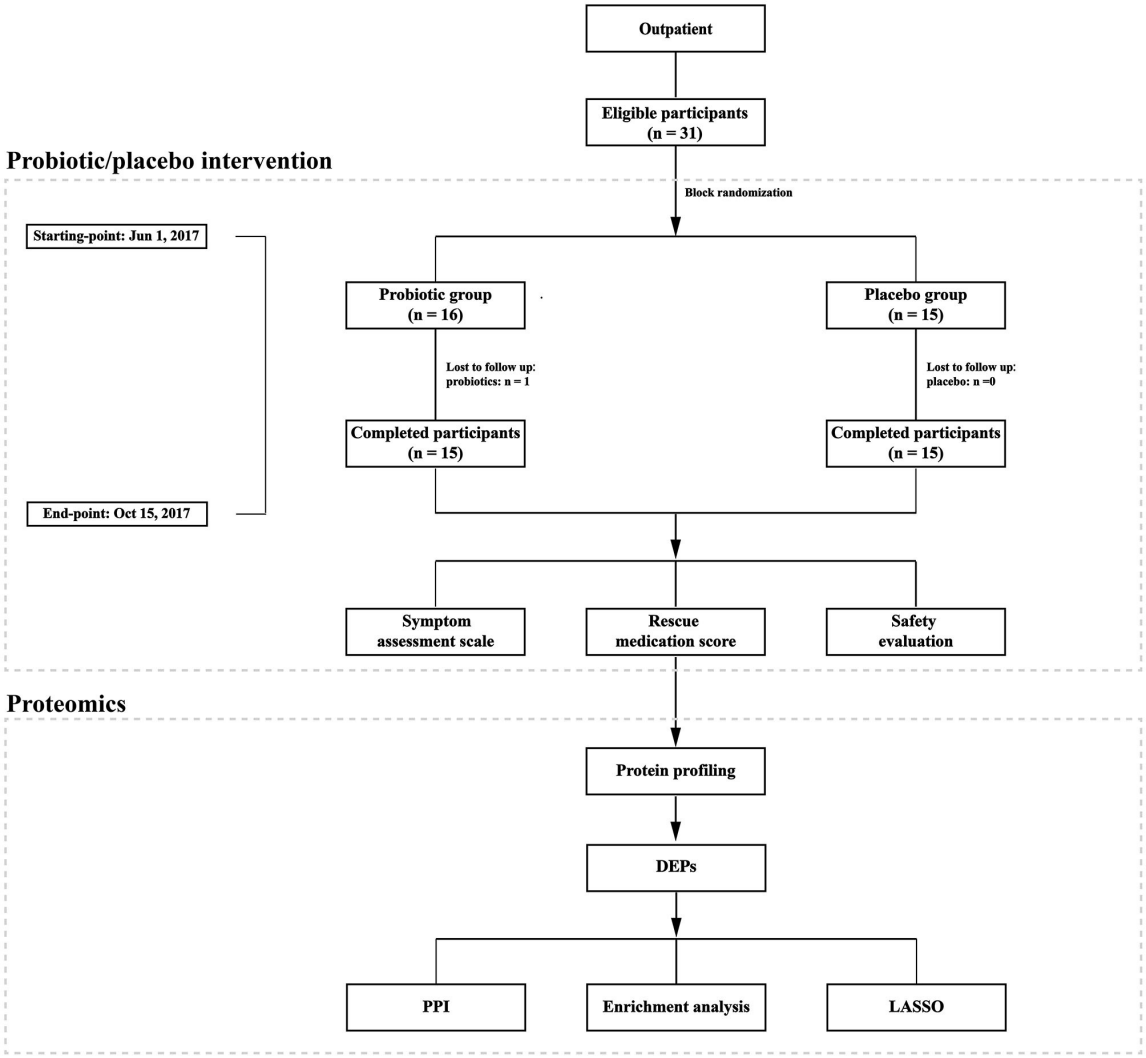
Schematic representation of the study design. A total of 31 eligible patients were enrolled in the study. They were randomly assigned into two groups to receive probiotic or placebo treatment. One participants dropped out in the placebo group. After completing the clinical trial, 12 participants' blood samples were used for further analysis. DEPs, differentially expressed proteins; PPI, protein–protein interaction; LASSO, Least Absolute Shrinkage and Selection Operator.

They were given a uniform medication regimen to relieve symptoms during the pollen season. The routine medication involves oral antihistamines, nasal corticosteroids, and antihistamine eye drops. Participants were asked to record the medication score, symptom score, and adverse effects in the electronic daily diary. Blood samples were collected at two time points: before the probiotic or placebo intervention in May or June (T1) and during the peak of the pollen season when patients suffer from the most severe AR symptoms (T2). A portion of the blood samples was randomly selected and processed for later protein profiling analyses. The whole landscape of the study is presented in Figure 1.

### Measurement

The visual analog scale (VAS score) provides details about the baseline severity of AR. The VAS was assessed by an allergist, and the score was based on rhinitis symptoms in the past pollen seasons. The score ranged from 0 (no discomfort) to 10 (maximal discomfort).

The symptom assessment scale (rhinoconjunctivitis symptom score) evaluates the frequency and severity of nasal and eye symptoms. The 4-point severity scale is presented in Table 1. The daily symptom score was the sum of all patients' symptom scores per day, ranging from 0 to 15.

**Table 1:** Symptom assessment scale.

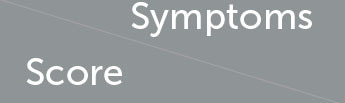	**Nasal itching**	**Nasal sneezing (times/day)**	**Nasal rhinorrhea (times/day)**	**Nasal stuffy nose**	**Eye itching**
0	None	< 3	0	None	None
1	Intermittent occurrence	3 – 9	≤ 4	Sometimes	Intermittent occurrence
2	Crawling sensation but bearable	10 – 14	5 – 9	Often	Strong feeling but bearable
3	Crawling sensation but unbearable	≥ 15	≥ 10	Keeping mouth breathing all day	Persistent strong feeling and unbearable

0 (absent): patients with rare symptoms, 1 (mild): symptoms were not troublesome, 2 (moderate): symptoms were troublesome but not disabling or unbearable, 3 (severe): symptoms were severe, disabling, and/or unbearable.

Rescue medication score evaluates the alleviating effect generated by taking medicine. Considering the different effects of anti-inflammatory drugs, they are assigned different scores. For example, oral antihistamine, 6 points per tablet; nasal corticosteroid, 1 point per spray; antihistamine eye drop, and 0.5 points per drop. The daily medication score was the sum of all subjects' medication scores per day.

### Pollen counts

The pollen counts were evaluated through gravity sedimentation as previously reported (Candeias et al., 2021). The pollen granule-capture device was installed at Peking Union Medical Hospital, which was in the center of Beijing, China. Pollen counts were documented from August 1, 2017 to September 30, 2017 and were presented as the number of grains per 1,000 square millimeters.

### PBMCs isolation and proteomic sample preparation

PBMCs were isolated using Ficoll density gradient centrifugation, as previously described (Boyum, 1964). After washing twice with phosphate buffer saline (PBS), cells were lysed with 8M Urea in PBS, 1% Protease Inhibitor Cocktail tablet (Roche), and 1 mM phenylmethanesulfonyl fluoride (PMSF). Protein concentrations were measured by BCA assay (Thermo Fisher Scientific). Each sample was diluted to 1 mg/ml for subsequent tandem mass tag (TMT) labeling.

In total, 200 μg of protein from each sample was used to conduct labeling. Protein extract was reduced with 5 mM dithiothreitol (DTT) at room temperature for 1 h and alkylated with 12.5 mM iodoacetamide (IAM) for 1 h in the dark. Samples were diluted with PBS, followed by trypsin digestion at a 100:1 protein/enzyme ratio overnight. The digested samples were desalted by Sep-Pak C18 columns (Waters, MA). Peptides from different samples were labeled with TMT reagents (Thermo Fisher Scientific), according to the manufacturer's instructions. Labeling reactions were quenched by using 5% hydroxylamine. All the TMT-labeled peptides from different samples were mixed and desalted by a Sep-Pak column.

Offline separation was conducted by a UPLC3000 system (Thermo Fisher Scientific) with an XbridgeTM BEH300 C18 column (5 μm, 150 Å, 250 mm × 4.6 mm i.d., Waters, MA). In brief, mobile phase buffer A is 2% acetonitrile and phase B is 98% acetonitrile. Both mobile phase buffers were adjusted by ammonium hydroxide to pH 10. The solvent gradient was set as follows: 8–18% phase B, 30 min; 18–32% phase B, 32 min; 32–95% phase B, 2 min; and 95% B, 6 min. Peptides were monitored at 214 nm and were collected. The collected fractions were dried by speed vac, reconstituted into 12 fractions, and re-dissolved in 0.1% (v/v) formic acid.

### LC/MS-MS identification and quantification

A 120-min gradient elution separated peptides from each fraction at a flow rate of 0.250 μl/min with EASY-nLCII™integrated nano-High Performance Liquid Chromatography System (Proxeon, Denmark). A Thermo Orbitrap Fusion Lumos mass spectrometer was used. The analytical column was a fused C-18 silica capillary column (75 μm ID, 150 mm length; Lexington, MA). Mobile phase A consisted of 0.1% formic acid, and mobile phase B consisted of 100% acetonitrile and 0.1% formic acid. The Orbitrap Fusion Lumos mass spectrometer was operated in the data-dependent acquisition mode. The scan range was from 350 to 1,550 m/z with 120,000 resolution with automatic gain control (AGC) target value of 2e5. A data-dependent acquisition method was performed to collect MS/MS spectra at 30,000 resolution and a maximum injection time (IT) of 60 ms for the top 20 ions observed in each mass spectrum. The exclusion duration was 8 s, and the collisional energy was 35%.

### MS data processing and analysis

The MS/MS spectra were searched against the UniProt Human database using the SEQUEST searching engine in Proteome Discoverer software version 2.1. Parameters for database searching were set as full tryptic specificity, and one missed cleavage was allowed. Carbamidomethylation (C) and TMTsixplex (K- and N-terminal) were set as fixed modifications. The oxidation (M) was set as variable modification. Precursor ion mass tolerances were set at 10 ppm for all MS acquired, and the fragment ion mass tolerance was set at 20 mmu for MS2 spectra acquired. The peptide spectral matches (PSMs) were validated at a 1% false discovery rate (FDR) under the percolator of the Proteome Discoverer software. Quantification was performed only for proteins with two or more unique peptide matches. The original proteomic data were taken pre-processing, such as removing outliers, filling missing values, and monitoring batch effect through R software.

### Statistical and bioinformatics analyses

The results were expressed as “mean ± standard deviation (SD)” if the parameters complied with a normal distribution or as “median [P_25_, P_75_]” if they presented as an abnormal distribution. Statistical analyses include paired-sample *t*-test and independent-sample *t*-test. All the above statistical analyses were computed using SPSS statistical software and R software.

For bioinformatics analyses, the R package “clusterProfiler” was used to perform the Gene Ontology (GO) and Kyoto Encyclopedia of Genes and Genomes (KEGG), while the R package “ggplot2” was applied to visualize the enriched pathways. PPI was constructed using the STRING online database (https://cn.string-db.org), and the network model was virtualized in Cytoscape (V3.9.1). CentiScaPe plugin was used to estimate the degree, betweenness, and closeness of nodes in the unweighted interaction network. The R package “glmnet” was used to conduct LASSO regression and variable selection was chosen using linear module strategy.

## Results

### Demographic characteristics of enrolled subjects

A total of 31 patients suffering from autumn seasonal AR were recruited, who were then randomly allocated into the probiotic group (*n* = 16) or placebo group (*n* = 15). As a participant missed multiple doses of probiotics, it was deemed as dropping out of the trial, and related recordings were not enrolled in later statistical analyses. There were 30 subjects, including 20 men and 10 women, who seriously followed the requirement and completed the clinical trial. Their age ranged from 23 to 62 years. Moreover, there was no statistically significant difference between the two groups in any aspects of the demographic and clinical features (Table 2). Notably, EOS% presented a statistical significance between the two time points (T1 and T2) in the placebo (*P* = 0.001) group while not presented in the probiotic group (*P* > 0.05). Although EOS% altered dramatically in comparison to the two groups, there was no statistical significance indicated by the independent-sample *t*-test. A detailed description is presented in Supplementary material 1.

**Table 2:** Baseline information about the probiotic group and the placebo group.

**Indices**		**Probiotic group (*n* = 15)**	**Placebo group (*n* = 15)**	***P***
Age (mean ± SD)		38.93 ± 10.95	39.00 ± 7.58	0.99
Sex (female: male)		2:1	2:1	1
Total IgE (KUA/L) (mean ± SD)		123.27 ± 99.02	78.44 ± 63.29	0.49
Eosinophils (× 10^9^ cells/L) (mean ± SD)	T1 time point	0.22 ± 0.10	0.23 ± 0.13	0.81
	T2 time point	0.69 ± 0.54	0.48 ± 0.22	0.31
EOS% (mean ± SD)	T1 time point	3.69 ± 1.71	3.60 ± 2.06	0.90
	T2 time point	3.69 ± 1.71	7.08 ± 2.02	0.24
Basophils (× 10^9^ cells/L) (mean ± SD)	T1 time point	0.03 ± 0.15	0.03 ± 0.02	0.87
	T2 time point	0.04 ± 0.02	0.04 ± 0.02	0.78
BASO% (mean ± SD)	T1 time point	0.43 ± 0.25	0.40 ± 0.23	0.75
	T2 time point	0.69 ± 0.54	0.56 ± 0.28	0.44
Lymphocyte (× 10^9^ cells/L) (mean ± SD)	T1 time point	1.74 ± 0.77	2.02 ± 0.49	0.25
	T2 time point	1.83 ± 0.46	1.83 ± 0.48	
LYM% (mean ± SD)	T1 time point	30.16 ± 8.55	31.12 ± 6.33	0.74
	T2 time point	28.35 ± 5.34	27.76 ± 6.03	0.79

EOS%, percentage of eosinophils; BASO%, percentage of basophils; LYM%, percentage of lymphocytes.

### Alleviating effect of administering probiotics on AR patients

**Figure 2:**
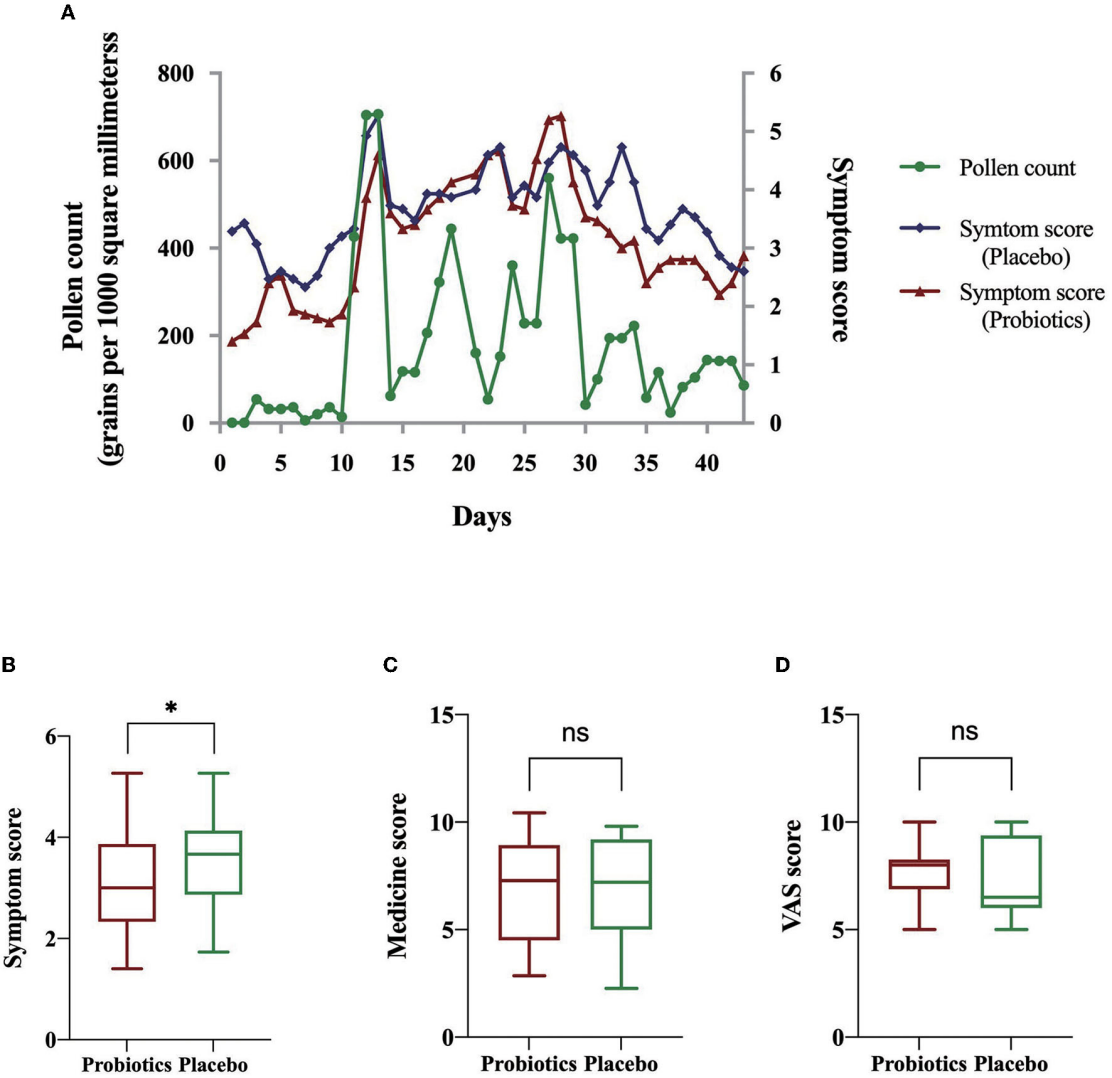
Amelioration effect evaluated by symptom score, rescue medicine score, and VAS score. **(A)** Daily pollen counts during pollen season and symptom scores of AR subjects in various groups. **(B)** Independent-sample *t*-test of symptom scores. **(C)** Independent-sample *t*-test of rescue medicine scores. **(D)** Independent-sample *t*-test of VAS scores. *informs *P* < 0.05; ns informs *P* > 0.05.

In China, autumnal pollen spores and grains generally spread from August to October (Yao and Zhang, 2009; Li et al., 2015). Therefore, we monitored the pollen grain concentrations to define “pollen season” in 2017. According to the EAN definition of pollen season (Bastl et al., 2015, 2018), the autumn allergy season lasted for 43 days in Beijing, China, starting on 13 August 2017. The median is 116 (36, 228) grains per 1,000 square millimeters. Moreover, the peak of pollen concentration was from the last 7 days of August to the first 14 days of September (Figure 2A). Correspondingly, the clinical manifestations were more prominent at the end period of August and the onset period of September as indicated by the daily recorded symptom assessment scales (Figure 2A). The average rhinoconjunctivitis symptom score of the probiotic group (3.11 ± 1.03) was significantly lower than that of the placebo group (3.54 ± 0.85, *P* = 0.037) during the pollen season (Figure 2B). The correlation analysis between pollen counts and clinical manifestations was then performed, and the correlation coefficient showed that these two factors were highly related (0.742 in the placebo group and 0.864 in the probiotic group).

Considering that the alleviated symptoms in the probiotic group may be caused by pharmaceutical intervention or baseline severity of the AR, we, then, analyzed medication usage and the initial AR severity by calculating medicine score and VAS score. The medicine scores and VAS scores were not statistically significantly different between the two groups (*P* > 0.05; Figures 2C, D). The above results suggested that the ameliorating effect from probiotics was not caused by pharmaceutical intervention or baseline symptom severity. A more detailed description is presented in Supplementary material 2.

### Safety evaluation

During the probiotic mixture or placebo administration period, no adverse event was reported. Just one participants in the probiotic group (1/15, 6.7%) experienced diarrhea after eating probiotic. However, the symptom was mild and relieved quickly without interrupting the continuous use of probiotics. Moreover, one subject in the placebo group also experienced diarrhea (1/15, 6.7%). No other adverse drug reactions (e.g., nausea, vomiting, fever, abdominal pain, and hematochezia) were observed, and none of the patients dropped out because of side effects.

### Identification of differentially expressed proteins (DEPs) responsible for AR and subsequent bioinformatics analyses

**Figure 3:**
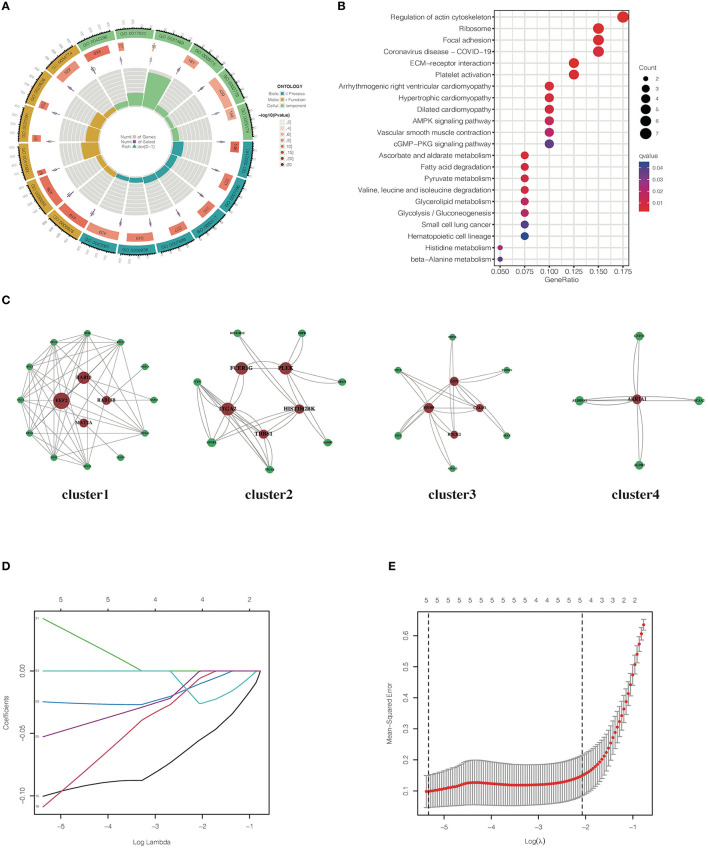
Bioinformatics analyses of DEPs identified in placebo group. **(A)** GO enrichment analysis of DEPs screened in the placebo group. **(B)** KEGG enrichment analysis of DEPs screened in the placebo group. **(C)** Co-expression network of DEGs identified in the placebo group. **(D)** LASSO coefficient profile of DEGs identified in the placebo group. **(E)** The misclassification error of LASSO in the jackknife rates analysis.

A total of six participants were randomly sampled from the placebo group, and the whole blood was collected to isolate PBMCs for further proteomics. DEPs were, then, screened following the criteria: (i) ≥ 95% confidence level, (ii) the number of matching peptides ≥ 2, (iii) fold change > 1.2 or < 0.83, and (iv) false discovery rate (FDR) < 0.05. A total of 12,448 proteins were identified with 967 proteins statistically expressed in all samples (Supplementary material 3). There were 60 DEPs, 35 downregulated and 25 upregulated, screened in patients with placebo administration when comparing T1 and T2 (Supplementary material 4), reflecting the pathogenic condition of AR. In total, 60 DEPs, then, underwent GO and KEGG enrichment analyses. GO analysis informed that these DEGs mainly are enriched in the coagulation process, transcription and translation, cell phagocytosis, and nutrient metabolic pathways (Figure 3A). The KEGG enrichment result showed that the regulation of the actin cytoskeleton, ribosome activity, platelet activation, inflammatory signal pathways, lipid metabolism, and amino acid metabolism was significantly enriched (Figure 3B). PPI co-expression network highlighted functionally interacted proteins. The network, including four separate clusters, contains 60 nodes and 82 edges. To obtain key proteins that play dominant roles, the network was then analyzed by the CentiScaPe plugin to calculate the degree, betweenness, and closeness between nodes. Key proteins were, then, filtered in various clusters and marked red (Figure 3C). To identify AR signature proteins, LASSO regression was applied to extract the main elements correlated with AR symptoms. In this study, the response type was set as Gaussian, and the alpha was set to 1. Figure 3D describes the coefficient profile of six indicators, which are generated using five-fold cross-validation. Figure 3E presents 10-fold cross-validation for tuning parameter selection in the model via minimum criteria. As a result, glutathione S-transferase omega-1 (GSTO1) (coeff. = −0.0998), sarcoplasmic/endoplasmic reticulum calcium ATPase 2 (ATP2A2) (coeff. = −0.107), minichromosome maintenance complex component 7 (MCM7) (coeff. = 0.0409), vitamin K-dependent protein S (PROS1) (coeff. = −0.0248), and E3 ubiquitin-protein ligase TRIM58 (TRIM58) (coeff. = −0.0522) were screened (Figures 3D, E).

### Comparison of DEPs in two groups to reveal potential targets of the novel probiotics

**Figure 4:**
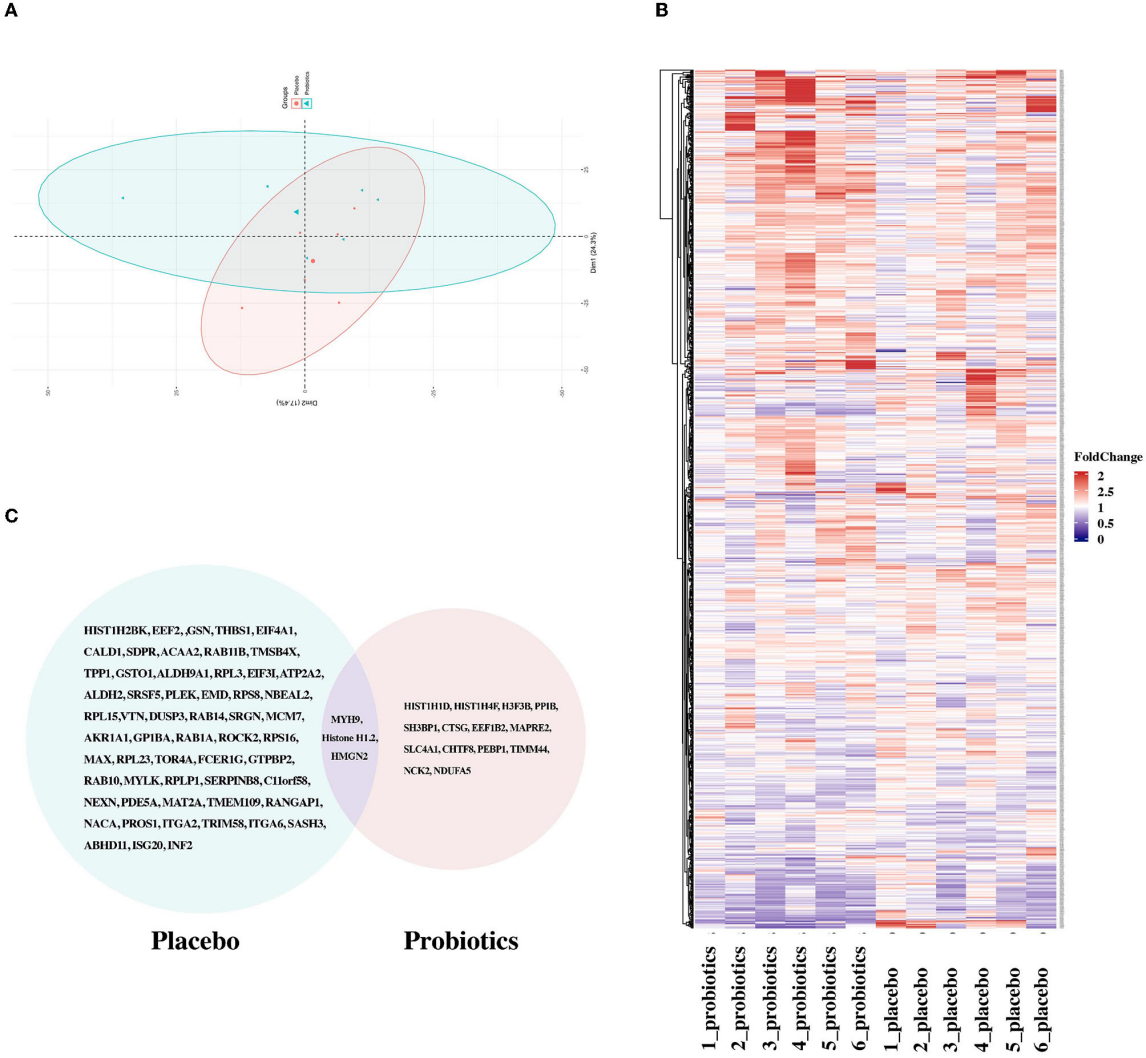
Comparison of DEPs between the probiotic group and placebo group. **(A)** Principal component analysis implied segregation and aggregation among samples. **(B)** Heatmaps of proteins identified in the probiotic and placebo groups. **(C)** Venn image of DEPs screened in the two groups.

To explore the potential targets of probiotics, six samples were randomly assigned from the probiotic group for further proteomic analyses. In a comparison of the time points of T1 and T2, 17 DEPs, composed of 6 downregulated and 11 upregulated proteins, were obtained in the probiotic group. The schematic landscapes of DEPs were notable differences in the two groups as indicated by principal component analysis and complex heap map (Figures 4A, B). There were only three common DEPs (MYH9, HMGN2, and Histone H1.2) in both groups (Figure 4C). Different from MYH9, whose expression levels were identical in two groups, HMGN2 and Histone H1.2 exist a significant reverse trend in groups with different intervention methods (Supplementary material 4).

## Discussion

Allergic rhinitis remains a constant challenge worldwide, and there was an urgent need to explore new effective treatment methods. Recently, probiotic intervention showed clinical benefits in various diseases by mediating immune function and presents a promising alternative for AR. Our research examined a novel probiotic mixture in alleviating AR-related clinical manifestations through a randomized, placebo-controlled, double-blind study. Furthermore, to explore the immunological mechanisms underlying probiotic intervention, TMT-based proteomic profiling was performed, and subsequent bioinformatics analyses were conducted.

Our results indicated that the novel multi-strain probiotics could effectively decrease EOS% without affecting other blood cells. The EOS% level is reported to be elevated in patients suffering from AR and the index can help to diagnose AR. In addition, the probiotic mixture could effectively alleviate AR clinical manifestations, and the symptom amelioration is independent of pharmaceutical therapy and the baseline severity, according to symptom scores, rescue medicine scores, and VAS scores. Moreover, the symptom loads were positively correlated with airborne pollen levels, which is in accordance with previous studies (Karatzas et al., 2018).

In this study, if the specific symptom was considered separately, significant decreases could be found in three nasal symptom scores, including sneezing, rhinorrhea, and nasal itching score in the probiotic group compared with the placebo group. However, no marked differences were identified in scores for eye itching between the two groups. Two studies (Xiao et al., 2006a; Gotoh et al., 2009) were conducted on Japanese AR patients who were allergic to cedar pollens and also had similar findings to our study. They reported that nasal symptom scores were significantly lower in the probiotic group than the control group while ocular scores did not differ between the two groups. Moreover, rescue medicines were requisite in almost all the literature reports mentioned above, indicating that probiotics alone could not completely control AR symptoms without the combined action of the symptomatic medication. Until now, probiotics still play an assistant role in the systemic treatment of AR, and further exploration is needed to develop novel probiotic products with a stronger effect.

The proteomic analysis identified a number of DEGs that may be involved in the development of AR, including chromosomal proteins, ribosomal proteins, and proteins mediating inflammation pathways. The GO and KEGG enrichment analyses supported the former and identified molecular signaling pathways in the pathophysiology of AR. For example, the inflammation pathways, such as the cGMP-PKG signaling pathway (Zhang X. et al., 2022) and AMPK signaling pathway (Zhang et al., 2022a), coagulation cascade (Hong et al., 2018), lipid, carbohydrate, and amino acid metabolic pathways (Gadzhimirzaev et al., 2011; Sun et al., 2021), transcription and translation processes, were reported to contribute to the initiation and exacerbation of AR. A total of 14 hub proteins, elongation factor 2 (EEF2), Ras-related protein Rab-10 (RAB10), Ras-related protein Rab-11B (RAB11B), S-adenosylmethionine synthase isoform type-2 (MAT2A), high-affinity immunoglobulin epsilon receptor subunit gamma (FCER1G), integrin alpha-2 (ITGA2), pleckstrin (PLEK), thrombospondin-1, (THBS1), histone cluster 1 h2b family member k (HIST1H2BK), gelsolin (GSN), caldesmon 1 (CALD1), rho-associated protein kinase 2 (ROCK2), myosin heavy chain 9/10/11/14 (MYH9), and alcohol dehydrogenase [NADP(+)] (AKR1A1), may play a pivotal role in the pathology of AR. These proteins are mainly chromosomal proteins, indicating that the main events in the development of AR may occur in the chromosome. Moreover, GSTO1, ATP2A2, MCM7, PROS1, and TRIM58 show great potential to be AR biomarkers as indicated by machine learning.

After the probiotic intervention, the expression level of non-histone chromosomal protein HMG-17, also named as the high-mobility group nucleosome-binding domain-containing protein 2 (HMGN2), elevated significantly, while Histone H1.2 present a sharp decrease. This informs that the novel probiotics moderate AR symptoms simply by targeting HMGN2 and Histone H1.2. HMGN2 is a non-histone chromosome protein, which is abundantly and ubiquitously expressed in the nuclei of higher eukaryotes (Srikantha et al., 1987; Landsman et al., 1989). Non-histone chromosomal proteins play a critical role in maintaining the structure of the chromatins and regulating chromatin-templated biological processes. For example, the high-mobility group nucleosome-binding (HMGN) proteins, a family of structural non-histone chromosomal proteins, destabilize higher-order chromatin structures to modulate the binding of nuclear regulatory factors, resulting in the regulation of gene expression and DNA replication (Crippa et al., 1992). Histone H1.2 is a variant of the linker histone H1, which serves as an intranucleosomal architectural protein and binds at the nucleosome entry and exit sites in a dynamic manner to form higher-order chromatin structures (Li et al., 2018; Höllmüller et al., 2021). HMGN2 has been reported to compete with histone H1 for binding to chromatin, blocking the chromatin-condensing activity of H1 (Postnikov and Bustin, 2016). Moreover, this type of competition may account for the opposite trend of these two proteins in this study (Ding et al., 1997). According to previous studies, the interaction of HMGN2 and Histone H1 could be triggered by the processes of ligand binding of the polypeptide hormone prolactin (PRL) and PRL receptor (PRLr) and the subsequent STAT5 activation (Schauwecker et al., 2017). Upon RPL-RPLr ligand binding, HMGN2 is recruited to the promoter of a STAT5 target gene, cytokine-inducible SH2-containing protein (*CISH*) gene, inducing the loss of both the linker histone H1 and nucleosome core particles. This process induces chromatin decompaction at the promoter *CISH*, allowing the recruitment of STAT5 (Reynolds et al., 1997; Yamashita et al., 2001). STAT5 serves as a critical factor in the modulation of T cell differentiation and function in the pathogenesis of AR (Zhang et al., 2022b). In sum, the novel probiotics achieve alleviating effects by elevating the expression level of HMGN2 and lowering the expression level of Histone H1. This event triggers *CISH* chromanone decompaction and the subsequent recruitment of STAT5. STAT5 regulates T-cell function and differentiation to confer clinical benefits for AR subjects.

There are some limitations to this research. For instance, the small sample size may introduce sample and selection bias. Thus, future clinical trials with a larger sample size should be performed to confirm the benefit of this multi-strain probiotic mixture in treating allergic airway diseases. Additionally, further research is required to certify and understand the regulatory role of HMGN2 and Histone H1.2 in seasonal AR.

## Conclusion

This study showed that pollen-induced AR was effectively ameliorated by treatment with the novel multi-strain probiotic mixture. Further proteomic analyses suggested the alleviating effect may be achieved by targeting HMGN2 and Histone H1.2.

## Data availability statement

The original contributions presented in the study are publicly available. This data can be found in the iProX - integrated Proteom resources database under accession number: (IPX0007002000).

## Ethics statement

The studies involving human participants were reviewed and approved by the Medical Ethics Committee of the Peking Union Medical College Hospital (JS-1649). The patients/participants provided their written informed consent to participate in this study.

## Author contributions

LL: investigation, data curation, and writing original draft. XW: formal analysis, data curation, and writing original draft. YG, YC, and JX: resources and investigation. JS, HD, and KG: conceptualization, supervision, and writing reviewing and editing.
